# The effect of plasma-treated nutrient solution on yield, pigments, and mineral content of rocket [*Diplotaxis tenuifolia* (L.) DC.] grown under different nitrogen fertilization levels

**DOI:** 10.3389/fpls.2024.1511335

**Published:** 2024-12-23

**Authors:** Mirko Sodini, Silvia Traversari, Sonia Cacini, Irene Gonfiotti, Anna Lenzi, Daniele Massa

**Affiliations:** ^1^ Department of Agricultural, Food, Environmental and Animal Sciences, University of Udine, Udine, Italy; ^2^ Research Institute on Terrestrial Ecosystems (IRET), National Research Council (CNR), Pisa, Italy; ^3^ Research Centre for Vegetables and Ornamental Crops, Council for Agricultural Research and Economics (CREA), Pescia (PT), Italy; ^4^ Department of Agriculture, Food, Environment and Forestry (DAGRI), University of Florence, Florence, Italy

**Keywords:** anthocyanins, biomass, cold plasma, *Diplotaxis tenuifolia*, nutraceutical value, sustainable horticulture

## Abstract

**Introduction:**

The non-thermal plasma (NTP) technique has been suggested as a sustainable horticultural practice to promote biomass accumulation, nutrient uptake, N metabolism, and disease prevention in plants. In particular, the potentiality of this technique to promote the natural accumulation of nutrients into plants deserve to be explored as input saving is strongly recommended in the horticultural sector.

**Methods:**

The nutrient solution supplied to a red coloured variety of rocket salad [*Diplotaxis tenuifolia* (L.) DC. ‘Dragon’s Tongue’] grown in a hydroponic close loop system was treated with NTP. Low, medium, and high concentrations of N (i.e., 1, 10, and 20 mM) of the nutrient solution were tested in control (no NTP) or NTP treated conditions in two consecutive growing cycles.

**Results and discussion:**

Results highlighted a N-dependent effect of NTP treatment showing a biomass stimulation at 10 mM N while negative effects of this technique at 1 and 20mM N. The biomass boosting of NTP found at 10 mMN coupled with an increase in K and Zn showing positive effects also on the nutraceutical aspects. Interestingly, different mechanisms seemed to be involved in the detrimental effects found at low and high N levels, i.e., a lower sensibility to N deficiency at 1 mM and a synergic negative effect of N and NTP in promoting oxidative stress at 20 mM.

## 1 Introduction

Nitrogen (N) is an essential element for all organisms, being a component mainly of proteins, amino acids, nucleic acids, membrane lipids, ATP, NADH. In plants, N promotes growth and development and is considered as a driving force for crop yield (Anas et al., 2020; Liu et al., 2022). This element is mainly absorbed from soil by the roots as nitrate or ammonium, although plants can absorb N from the leaves when applied by foliar spray (Xu et al., 2012). An adequate N availability in the root zone is crucial to obtain high yields (Tei et al., 2020). On the other hand, excessive N in cropping systems poses environmental hazards and may cause adverse effects on the crops themselves. From an environmental point of view, the main concerns are water eutrophication, soil acidification, and high rate of greenhouse gas emissions (Anas et al., 2020; Zamanian et al., 2024). The detrimental effects on crops include increased susceptibility to pathogen attacks (Sharma, 2020) and excessive vegetative growth (Leghari et al., 2016). Faster decay (Ierna et al., 2020) and deterioration of many quality parameters (Albornoz, 2016) are observed during postharvest. Nitrates, whose possible harmful effects on human health are known (Santamaria, 2006), are a major concern in leafy vegetable productions, especially for those species which are particularly prone to their accumulation (Cavaiuolo and Ferrante, 2014). Since excess nitrates accumulate in plant cell vacuoles, any factor affecting uptake and/or assimilation modulates their concentration in plant tissues.

The Non-Thermal Plasma (NTP) technology applied to crops can affect N metabolism in plants, including its accumulation (Brar et al., 2016; Peethambaran et al., 2015). The term “plasma” refers to a totally or partially ionized gas, composed of several chemically active components (Nehra et al., 2008). The NTP, belonging to plasmas artificially obtained by applying any form of energy to induce gas ionization (Nehra et al., 2008; Phan et al., 2017), can find scientific and industrial applications in various fields, including medicine, electronics, material sciences, agriculture, and food industry (Konchekov et al., 2023). In particular, NTP is trailed as a powerful technique for reducing chemical contaminants in agricultural products (Herianto et al., 2023). The main advantages are: the possibility of working with air, as it is at atmospheric pressure, low operating temperatures, short treatment times, efficient use of energy, and minimal environmental impact (Cherif et al., 2023; Rajan et al., 2023). On the other hand, some drawbacks limit the use of NPT, such as possible adverse alterations in the physicochemical characteristics of the treated food, and the difficulty to cover the entire surface during the decontamination treatments (Herianto et al., 2023). On the contrary, Plasma Activated Water (PAW), which is generated by treating water with a plasma stream, seems a more flexible alternative than the gaseous form for applications in the agricultural sector (Ruamrungsri et al., 2023). When energy input is applied to the gas, reactive oxygen and nitrogen species (ROS and RNS) are generated such as hydrogen peroxide, ozone, nitrate, nitrite, nitric oxide, ammonium ions. These molecules can be delivered to the gas-liquid interface and thus enrich PAW composition (Zhou et al., 2020). Moreover, other chemical aspects are modified in PAW such as a lower pH and a higher electrical conductivity (EC) and redox potential (Thirumdas et al., 2018).

In agriculture, plasma shows potentiality for pest and disease control due to its antimicrobial activity, but can also exert positive effects on plants like stimulating seed germination, root development, and plant metabolism, enhancing tolerance to biotic and abiotic stresses, improving nutrient availability and uptake, and increasing overall crop productivity and quality (Bora et al., 2022; Cannazzaro et al., 2021a, 2021b; Konchekov et al., 2023; Puač et al., 2018; Traversari et al., 2021). Regarding abiotic stress, NTP treatment has been showed to improve crop resistance to salinity (Veerana et al., 2024) and heavy metals (Ghasemzadeh et al., 2022). Although the mechanisms of plasma-plant interactions are not yet well understood, the use of plasma could be assimilated to that of a biostimulant, since it appears to be involved in the activation of various plant growth regulators and stress response genes, the induction of systemic resistance, and the strengthening of antioxidant defence mechanisms (Konchekov et al., 2023). Furthermore, PAW, administered as irrigation water, can directly counter pathogens that might attack the roots (Brandenburg et al., 2019) or the epigeal part (Cannazzaro et al., 2021a), and have a fertilizing effect due to the capacity of capturing atmospheric N (Puač et al., 2018). Among other valuable effects on plant quality, a significant increase in plant pigments and reduction of nitrate concentration in leafy vegetables irrigated with PAW have been observed (e.g., Cannazzaro et al., 2021a). Therefore, rocket, who naturally accumulates nitrates at the high levels (Cavaiuolo and Ferrante, 2014), has been chosen as test plant in this work. In particular, the wild rocket [*Diplotaxis tenuifolia* (L.) DC.] variety ‘Dragon’s Tongue’ has been selected for its flavour attributed to the presence of high anthocyanin and other compounds in the leaves, especially in the main leaf veins, which are red in colour.

Despite the potential of plasma technology for crop management, most studies have focused on seed or post-harvest treatments, while application during the cultivation phase has been scarcely investigated. The main difficulties concern the dosage and intensity of treatments, which are subject to dynamic changes also as a results of growing environment conditions. The aim of this work was to evaluate the effect of the interaction between NTP and N fertilization on crop yield and quality. For this purpose, the NTP was used to treat the nutrient solution supplied to wild rocket plants grown hydroponically in a greenhouse combining plasma treatment with different levels of N in the nutrient solution.

## 2 Materials and methods

### 2.1 Plant material and treatments

**Figure 1:**
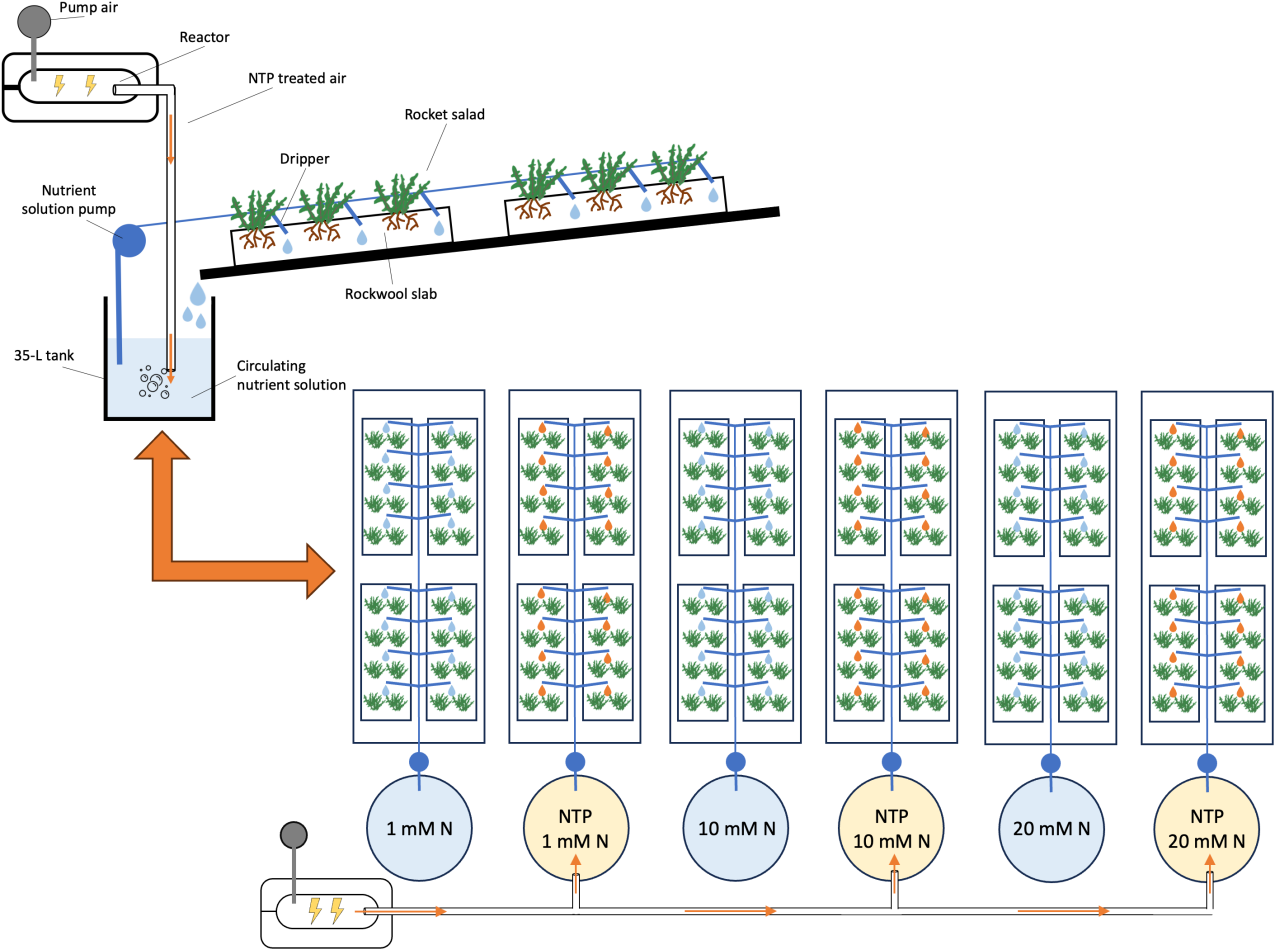
Description of NTP apparatus and experimental scheme.

The experiment was conducted in the experimental greenhouse described by Burchi et al. (2018), located at CREA Research Centre for Vegetable and Ornamental Crops in Pescia, Tuscany, Italy (lat. 43°54′ N, long. 10°42′ E), which is equipped with the NTP technology to treat the nutrient solution. Non-thermal plasma was generated by a Dielectric Barrier Discharge device (Jonix srl, Tribano, PD, Italy) set at 5-25 kV thereby producing 1012-1015 charged molecules per cm^–3^. Seeds of wild rocket salad [*Diplotaxis tenuifolia* (L.) DC. cv. ‘Dragon’s Tongue’] were sown on 3^rd^ November in alveolate trays filled with rockwool cylinders (3 seeds per cylinder), covered with vermiculite and irrigated with tap water. The trays were kept in a growth chamber till the emergence of seedlings (22°C, 75% relative humidity, 16:8 h of day:night photoperiod), and one plant (the strongest) per cylinder was left. Seedlings were transplanted at the four-true leaf stage in rockwool slabs (1 m × 0.15 m × 0.75 m) on 6^th^ December 2021 (8 units composed by a group of two plants per slab, thus 16 plants in total). The slabs were placed in the greenhouse on benches equipped with a drip irrigation system in closed loop system. Six nutrient solutions were then applied as different treatments (4 slabs per treatment, 24 slabs in total), as a combination of N concentration and NTP use: 1) 1 mM N without NTP; 2) 1 mM N with NTP; 3) 10 mM N without NTP; 4) 10 mM N with NTP; 5) 20 mM N without NTP; 6) 20 mM N with NTP. In the NTP treatments, the nutrient solution was prepared by a fertigation unit and stocked in 35-L tanks where the nutrient solution was continuously treated by bubbling NTP-treated air. The flux of NTP-treated air was kept constant at 2 L min^-1^ in all tanks, by measuring the entering air flux with an analogical fluxmeter. The following basic nutritive recipe was used modulating the N concentration for 1 (10, or 20) mM N, respectively: N-NO_3_ 1.0 (10.0, or 19.6) mmol L^-1^, N-NH_4_ 0.06 (0.65, or 1.30) mmol L^-1^, P-PO_4_ 1.0 mmol L^-1^, K 6.0 mmol L^-1^, Ca 3.5 (3.5, or 5.5) mmol L^-1^, Mg 1.0 (1.0, or 2.0) mmol L^-1^, Na 0.65 mmol L^-1^, S-SO_4_ 3.7 (1.5, or 1.1) mmol L^-1^, Cl 3.93 mmol L^-1^, Fe 40 µmol L^-1^, B 30 µmol L^-1^, Cu 1 µmol L^-1^, Zn 5 µmol L^-1^, Mn 5 µmol L^-1^, Mo 1 µmol L^-1^. The pH of nutrient solutions was daily monitored, throughout the cultivation period, and maintained at 6 by adding sulfuric acid when needed. Nutrient concentrations and EC (1.65, 1.74 or 2.57 dS m^-1^) were kept fairly constant by adding fresh nutrient solution at estimated uptake concentration. The same plants were cultivated for two consecutive production cycles, exploiting the ability of *D. tenuifolia* to re-grow after harvest and considering the 2^nd^ growing cycle as the standard to be marketed. The effects of the treatments were evaluated at the end of both production cycles. The experimental scheme and the description of NTP system are reported in **Figure 1**.

### 2.2 Plant physiological measurements and sampling

Plant photosystem II efficiency (F_v_/F_m_) was assessed by MINI-PAM fluorimeter (Heinz Walz GmbH, Effeltrich, Germany) for both the growing cycles after a 20 min dark adaption of leaves. Plants were sampled on the 26^th^ January 2022 (1^st^ production cycle, 12 weeks after sowing) and the 9^th^ March 2022 (2^nd^ production cycle, 18 weeks after sowing). At both sampling days, shoot fresh weight (FW) of all the plants of each replicate was determined (one replicate = one slab). Two samples of leaf disks collected from young and completely unfolded leaves (100 mg FW each) were stored at – 80 °C for leaf pigment analysis. Sub-samples of leaves were used for measuring leaf area by a leaf area meter (WinDIAS Image Analysis System, Delta-T Devices, Cambridge, UK). Fresh material was dried at 50 °C to determine the dry weight (DW). Specific leaf area (SLA) was calculated as the ratio between the leaf area and DW of sample used for the measure.

### 2.3 Pigment measurements

Chlorophyll *a*, *b*, and carotenoids were extracted in two technical replicates from leaf disk samples with MeOH (0.1 mL mg^–1^ FW) at – 20 °C, renewing the solution after 1 d for a total of 2 d. The extracts were measured using a spectrophotometer (Evolution™ 300 UV-Vis Spectrophotometer, Thermo Fisher Scientific Inc., MA, USA) and pigment concentrations were obtained following the procedure reported by Lichtenthaler and Buschmann (2001). Anthocyanins and flavonols were determined as described for chlorophylls and carotenoids in two technical replicates using a mixture 80:17.7:2.3 v:v:v of MeOH:dH_2_O:37%HCl for the extraction, following the procedure reported by Hradzina et al. (1982).

### 2.4 Quantification of nutrients

Oven-dried leaf samples were ground to a fine powder. An amount of 0.30 g from each sample was digested by a microwave using a HNO_3_:H_2_O_2_ mixture (5:2 v:v), following the EPA Method 3051a. The total K, Ca, Mg, Fe, Na, Mn, and Zn concentrations in plants of the 2^nd^ production cycle were determined using inductively coupled plasma spectrometry (ICP-OES 5900 Agilent, Santa Clara, CA, USA) while P-PO_4_ concentration was spectrophotometrically determined through the molybdenum blue method. The N concentrations in different chemical forms were determined in leaves derived from both production cycles. Kjeldahl N was quantified through the Kjeldhal-Tecator method. An amount of 0.25 g of dried powder from each sample was digested with 12 mL of H_2_SO_4_ and a Selenium Catalyst Tablet (VELP Scientifica, Usmate, MB, Italy) at 400°C. Digested samples were analysed by VELP-UDK127 apparatus (VELP Scientifica, Usmate, MB, Italy), adding 50 mL of 40% w/v NaOH. The distillate was collected in a conical flask with 4% w/v boric acid and bromocresol green-methyl red colour indicator and Kjeldahl N concentration was determined by titration with 0.1 N HCl. Nitrates (NO_3_) were quantified by the nitration of salicylic acid using the procedure reported by Cataldo et al. (1975) comparing the absorbance at 410 nm against a calibration curve with a nitrate standard solution (Merck KGaA, Darmstadt, Germany). Nitrites (NO_2_) were determined using the diazotization-coupling Griess reaction as reported by Merino (2009) at 540 nm against a calibration curve with NaNO_2_ (Merck KGaA, Darmstadt, Germany). All the results were expressed as mg kg^−1^ DW.

### 2.5 Statistical analysis

Data were tested for normal distribution using Shapiro-Wilk normality test and eventually transformed before the ANOVA. Data (n = 4 replicate of 16 plants) were analysed with a two-way ANOVA (NTP and N as independent variables) and a Tukey’s *post-hoc* test to assess significant differences (*P* ≤ 0.05, 0.01, and 0.001). The statistical analyses and graphs were performed with Prism 10 (GraphPad Software, Inc., La Jolla, CA, USA).

## 3 Results

### 3.1 Biometric parameters

**Figure 2:**
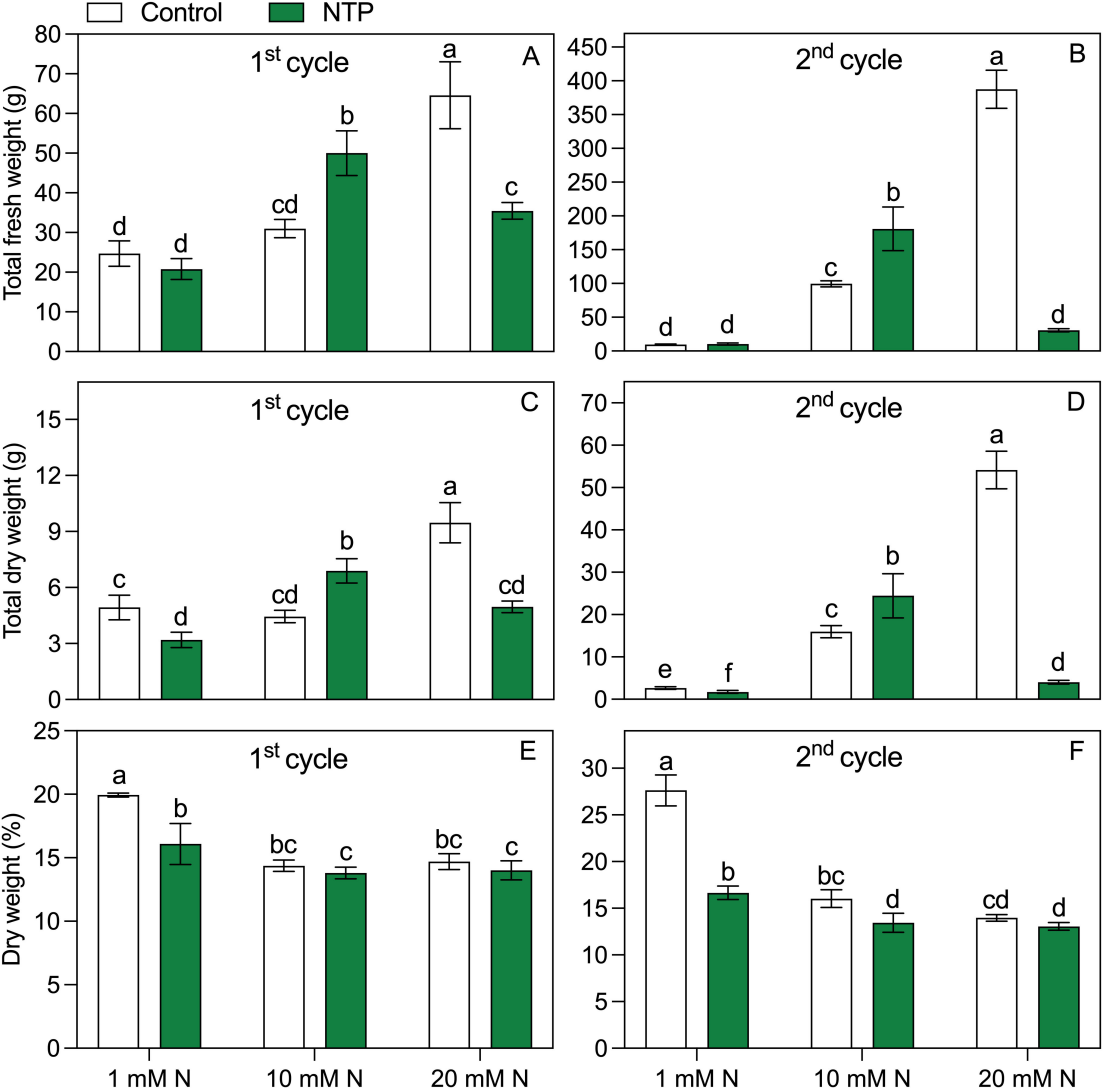
Total fresh **(A, B)** and dry **(C, D)** shoot weights and dry weight percentage **(E, F)** at the 1^st^ and 2^nd^ production cycle measured in rocket salad plants (total weight of 16 plants) grown using nutrient solution treated or untreated with NTP and with 1, 10, or 20 mM N. Bars represent mean values of four slabs ± SD. Pairwise comparison based on Tukey’s *post hoc* is shown in the figure as different letters when two-way ANOVA interaction (N and NTP as factors) was found significant considering separately the growing cycles (ANOVA *P*-values are reported in the **Table 1**).

Both factors (N level and NTP treatment) and their interaction showed a significant effect on biomass parameters (**Figure 2**; **Tables 1**, **2**). The fresh biomass had the highest value at 20 mM N and control (no NTP) conditions at both sampling times (**Figures 2A, B**). The NTP treatment showed a boosting effect on growth at 10 mM N concentration (+ 61% and + 82% at 1^st^ and 2^nd^ cycle, respectively). Conversely, NTP had no effect in plants at 1 mM N concentration and even caused a very strong biomass decrease at 20 mM N concentration, particularly evident at the 2^nd^ cycle (– 55% and – 92% at 1^st^ and 2^nd^ cycle, respectively). A similar trend was retrieved for the dry biomass (**Figures 2C, D**), but the NTP treatment significantly reduced this parameter also at 1 mM N concentration at both cycles. The dry biomass percentage (**Figures 2E, F**) was in general higher at 1 mM N concentration and was lower in NTP treated plants (– 20% and – 40% for the 1^st^ and 2^nd^ cycle at 1 mM N, respectively). The leaf area had the same trend as fresh biomass, too (**Table 2**). The specific leaf area reached the lowest value in control plants at 1 mM N concentration but under this N concentration the parameter increased when plants were treated with NTP (**Table 2**). Only slight variations were found in specific leaf area under other treatments.

**Table 1:** Two-way ANOVA (N and NTP as variables) *P*-values for biomass parameters (*P < 0.05; **P < 0.01; ***P < 0.001).

Parameter		Nitrogen (N)	NTP	N × NTP
Fresh weight	1^st^ cycle	***	*	***
	2^nd^ cycle	***	***	***
Dry weight	1^st^ cycle	***	***	***
	2^nd^ cycle	***	***	***
Leaf area	1^st^ cycle	***	**	***
	2^nd^ cycle	***	***	***
Specific leaf area	1^st^ cycle	***	**	*
	2^nd^ cycle	***	*	***
% Dry weight	1^st^ cycle	***	***	**
	2^nd^ cycle	***	***	***

**Table 2:** Leaf area and specific leaf area at the 1^st^ and 2^nd^ production cycle measured in rocket salad plants (total area of 16 plants) grown using nutrient solution treated or untreated with NTP and with 1, 10, or 20 mM N.

Parameter		1 mM N		10 mM N		20 mM N	
		Control	NTP	Control	NTP	Control	NTP
Leaf area (cm^2^)	1^st^ cycle	511 ± 80 cd	405 ± 44 d	605 ± 48 c	977 ± 114 b	1325 ± 148 a	637 ± 34 c
	2^nd^ cycle	169 ± 17 *d*	159 ± 35 *d*	1897 ± 120 *c*	3140 ± 662 *b*	6971 ± 509 *a*	518 ± 22 *d*
Specific leaf area (cm g^–1^)	1^st^ cycle	103 ± 5 d	127 ± 5 c	136 ± 3 abc	142 ± 6 a	140 ± 8 ab	128 ± 5 bc
	2^nd^ cycle	65 ± 12 *c*	91 ± 1 *b*	119 ± 6 *a*	130 ± 16 *a*	129 ± 2 *a*	129 ± 8 *a*

Values represent the means of four slabs ± SD. Pairwise comparison based on Tukey’s *post hoc* is shown in the table as different letters when two-way ANOVA interaction (N and NTP as factors) was found significant considering separately the growing cycles (ANOVA *P*-values are reported in **Table 1**).

### 3.2 Photosystem II efficiency and amounts of pigments

The photosystem II efficiency F_v_/F_m_ measurements (**Tables 3**, **4**) generally fell within the range of healthy plants; only at the 2^nd^ production cycle this parameter significantly lowered at 20 mM N in NTP treated plants. Chlorophyll *a* and *b* and carotenoids had similar trends (**Tables 3**, **4**) with NTP influencing their amounts only considering its interaction with N levels just at the 1^st^ growing cycle. On the contrary, N level affected pigment amount at the 2^nd^ growing cycle. The anthocyanin concentration (**Tables 3**, **4**) increased at 1 mM N and control conditions compared with all other treatments. At the 1^st^ production cycle, the amount of these molecules in control plants irrigated with 1 mM of N was 52% higher compared with the NTP treated plants with the same N level, while 77% higher at the 2^nd^ production cycle. The flavonol concentration was lower in NTP treated plants at 1 mM N compared to all other treatments at the 2^nd^ growing cycle (**Tables 3**, **4**).

**Table 3:** Two-way ANOVA (N and NTP as factors) *P*-values for photosystem II efficiency and pigment concentrations (*P < 0.05; **P < 0.01; ***P < 0.001; ns, not significant).

Parameter		Nitrogen (N)	NTP	N × NTP
F_v_/F_m_	1^st^ cycle	ns	**	ns
	2^nd^ cycle	**	***	*
Chlorophyll *a*	1^st^ cycle	*	ns	**
	2^nd^ cycle	***	ns	ns
Chlorophyll *b*	1^st^ cycle	ns	ns	**
	2^nd^ cycle	***	ns	ns
Carotenoids	1^st^ cycle	ns	ns	*
	2^nd^ cycle	***	ns	ns
Anthocyanins	1^st^ cycle	***	***	***
	2^nd^ cycle	ns	***	***
Flavonols	1^st^ cycle	*	ns	*
	2^nd^ cycle	ns	**	*

**Table 4:** Photosystem II efficiency and pigment concentrations at the 1^st^ and 2^nd^ production cycle measured in rocket salad plants grown using nutrient solution treated or untreated with NTP and with 1, 10, or 20 mM N.

Parameter		1 mM N		10 mM N		20 mM N	
		Control	NTP	Control	NTP	Control	NTP
F_v_/F_m_	1^st^ cycle	0.83 ± 0.02	0.81 ± 0.04	0.84 ± 0.01	0.82 ± 0.02	0.85 ± 0.01	0.79 ± 0.04
	2^nd^ cycle	0.83 ± 0.01 *a*	0.78 ± 0.05 *ab*	0.83 ± 0.02 *a*	0.83 ± 0.01 *a*	0.83 ± 0.01 *a*	0.75 ± 0.02 *b*
Chlorophyll *a* (mg g^–1^ DW)	1^st^ cycle	2.53 ± 0.50 b	3.96 ± 0.94 ab	4.05 ± 0.60 ab	3.79 ± 0.74 ab	4.04 ± 1.24 a	3.43 ± 0.79 ab
	2^nd^ cycle	1.28 ± 0.57	1.03 ± 0.37	4.92 ± 0.74	4.62 ± 1.77	4.74 ± 0.60	3.08 ± 2.47
Chlorophyll *b* (mg g^–1^ DW)	1^st^ cycle	0.84 ± 0.21 b	1.42 ± 0.39 ab	1.41 ± 0.17 ab	1.45 ± 0.53 ab	1.69 ± 0.17 a	1.22 ± 0.40 ab
	2^nd^ cycle	0.56 ± 0.17	0.60 ± 0.49	1.90 ± 0.39	1.83 ± 0.75	2.21 ± 0.13	1.71 ± 0.27
Carotenoids (mg g^–1^ DW)	1^st^ cycle	0.73 ± 0.08	1.11 ± 0.37	1.08 ± 0.16	0.88 ± 0.07	1.23 ± 0.33	0.91 ± 0.16
	2^nd^ cycle	0.42 ± 0.19	0.32 ± 0.06	1.41 ± 0.18	1.32 ± 0.38	1.21 ± 0.22	0.66 ± 0.77
Anthocyanins (mg g^–1^ DW)	1^st^ cycle	1.94 ± 0.10 a	0.92 ± 0.22 b	0.92 ± 0.09 b	0.98 ± 0.13 b	1.02 ± 0.20 b	0.96 ± 0.16 b
	2^nd^ cycle	1.42 ± 0.17 *a*	0.33 ± 0.15 *d*	0.93 ± 0.19 *bc*	0.66 ± 0.12 *cd*	1.06 ± 0.08 *b*	0.75 ± 1.81 *bc*
Flavonols (mg g^–1^ DW)	1^st^ cycle	7.00 ± 0.32 ab	7.81 ± 1.19 a	6.60 ± 0.66 ab	6.21 ± 0.57 ab	7.10 ± 0.71 ab	5.80 ± 0.80 b
	2^nd^ cycle	6.39 ± 0.82 *a*	3.39 ± 0.28 *b*	5.6 ± 1.35 *a*	5.4 ± 1.27 *ab*	5.59 ± 0.82 *a*	5.37 ± 0.79 *ab*

Values represent mean values (n = 4, in two technical replicates) ± SD. Pairwise comparison based on Tukey’s *post hoc* is shown in the table as different letters when two-way ANOVA interaction (N and NTP as factors) was found significant considering separately the growing cycles (ANOVA *P*-values are reported in **Table 3**).

### 3.3 Plant nutrients

**Figure 3:**
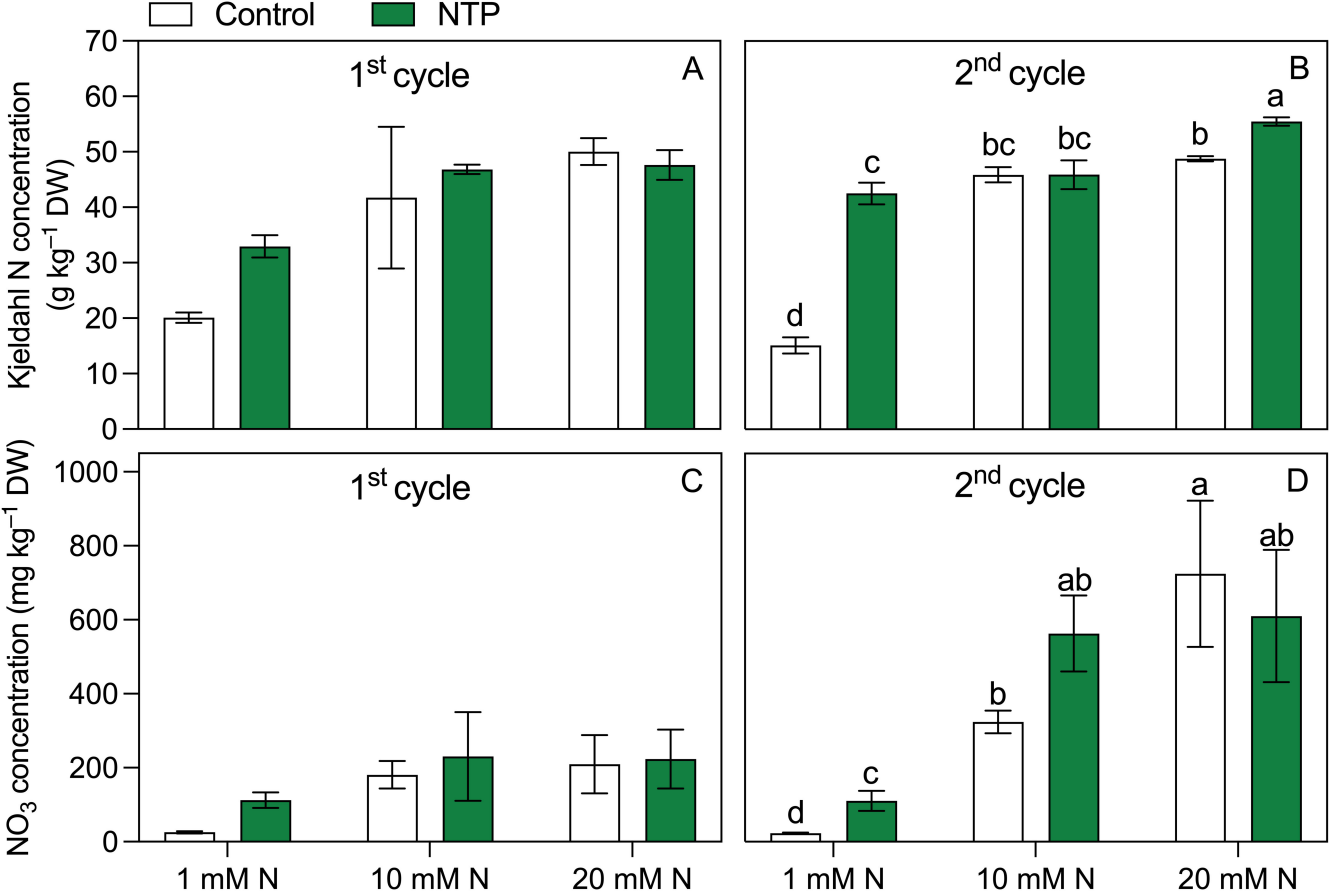
Kjeldhal N **(A, B)** and NO_3_ **(C, D)** concentrations at the 1^st^ and 2^nd^ production cycle measured in rocket salad shoot grown using nutrient solution treated or untreated with NTP and with 1, 10, or 20 mM N. Bars represent mean values (n = 4) ± SD. Pairwise comparisons based on Tukey’s *post hoc* are shown in the figure as different letters when two-way ANOVA interaction (N and NTP as factors) was found significant considering separately the growing cycles (ANOVA *P*-values are reported in **Table 5**).

*Kjeldahl* N (**Figures 3A, B**; **Table 5**) was influenced by N concentration at both sampling times but the effect of NTP treatment and the interaction between N and NTP was significant only at the 2^nd^ cycle. Notably, at the 2^nd^ growing cycle plants fed with 1 mM and 20 mM N had an increase in shoot *Kjeldahl* N concentration under NTP treatment compared to the untreated plants (+ 180% and +14%, respectively), particularly strong at 1 mM N. The NTP influenced the NO_3_ concentration in the 2^nd^ cycle (**Figures 3C, D**; **Table 5**) but this N form was statistically higher in NTP treated plants only at 1 mM N concentration (+ 380%). The NO_2_ concentration measured in the shoots was under the detection limit of the methodology at both sampling times and for all treatments (data not shown).

**Table 5:** Two-way ANOVA (N and NTP as variables) *P*-values of plant nutrients (*P < 0.05; **P < 0.01; ***P < 0.001; ns, not significant).

Parameter		Nitrogen (N)	NTP	N × NTP
Kjeldahl N	1^st^ cycle	*	ns	ns
	2^nd^ cycle	***	***	***
NO_3_	1^st^ cycle	***	ns	ns
	2^nd^ cycle	***	***	***
Ca	2^nd^ cycle	***	*	***
K	2^nd^ cycle	***	***	***
Mg	2^nd^ cycle	***	***	***
P-PO_4_	2^nd^ cycle	**	**	**
Fe	2^nd^ cycle	***	ns	***
Na	2^nd^ cycle	***	**	***
Zn	2^nd^ cycle	***	***	***
Mn	2^nd^ cycle	***	*	***

**Figure 4:**
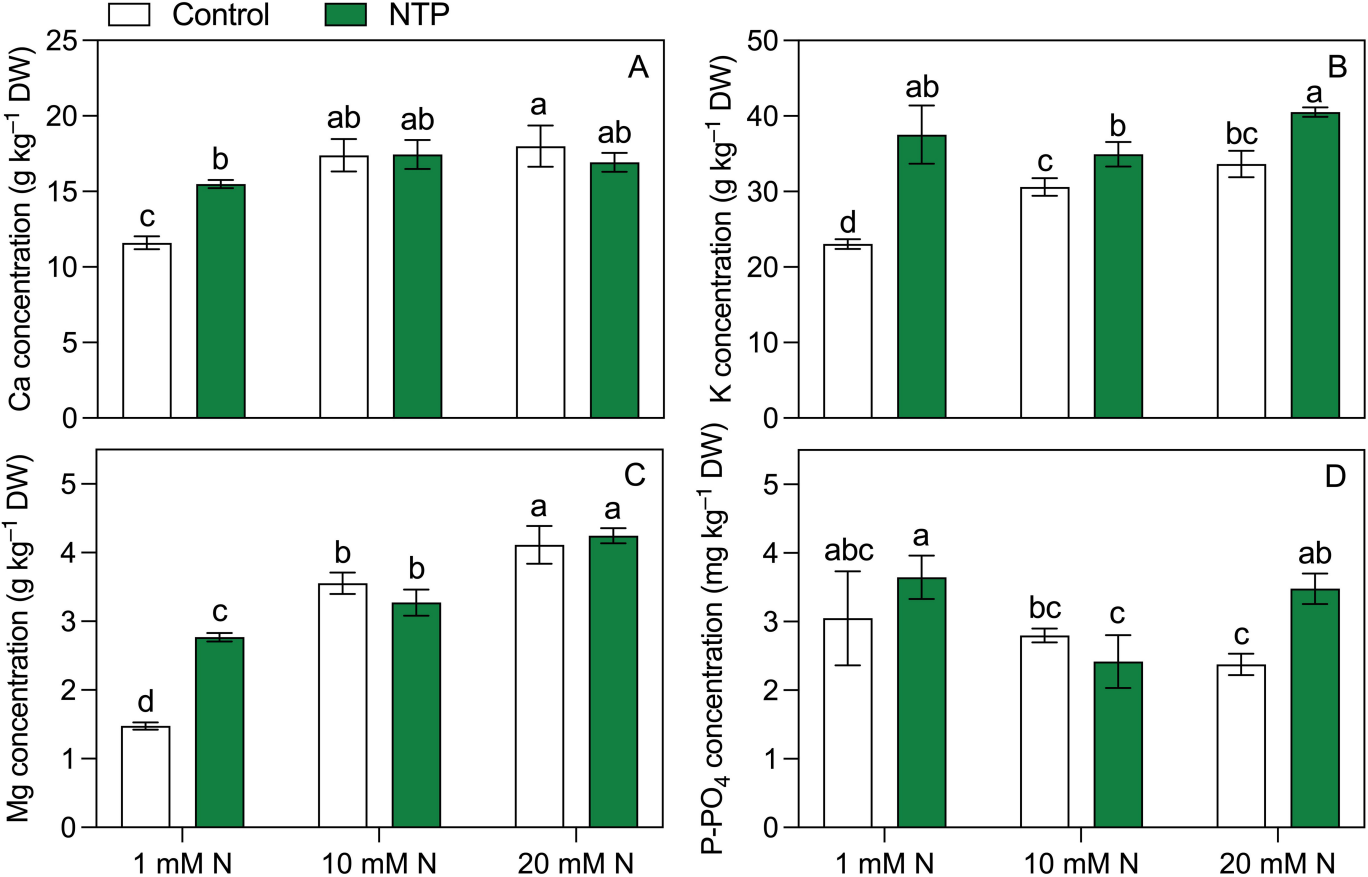
Ca **(A)**, K **(B)**, Mg **(C)**, and P-PO_4_ **(D)** concentrations at the 2^nd^ production cycle measured in rocket salad shoot grown using nutrient solution treated or untreated with NTP and with 1, 10, or 20 mM N. Bars represent mean values (n = 4) ± SD. Pairwise comparisons based on Tukey’s *post hoc* are shown in the figure as different letters when two-way ANOVA interaction (N and NTP as factors) was found significant (ANOVA *P*-values are reported in **Table 5**).

Other plant macronutrients measured at the 2^nd^ growing cycle (**Figure 4**; **Table 5**) were all influenced by the interaction between NTP treatment and N concentration in the nutrient solution. Calcium concentration (**Figure 3A**) was higher in 10 and 20 mM N treatments and it was higher in NTP treated plants at 1 mM N compared with the untreated plants (+ 33%). Potassium concentration (**Figure 3B**) was increased by both N concentration and NTP treatment. In particular, K concentration was 63%, 14%, and 20% higher in NTP treated plants at 1, 10, and 20 mM N, respectively. Magnesium concentration (**Figure 4C**) was increased by N level and was significantly higher in NTP treated plants at 1 mM N (+ 88%). Phosphorus concentration (**Figure 3D**) showed a different trend compared with the other macronutrients. It was lower in 20 mM control plants compared to the 1 and 20 mM NTP-treated plants.

**Figure 5:**
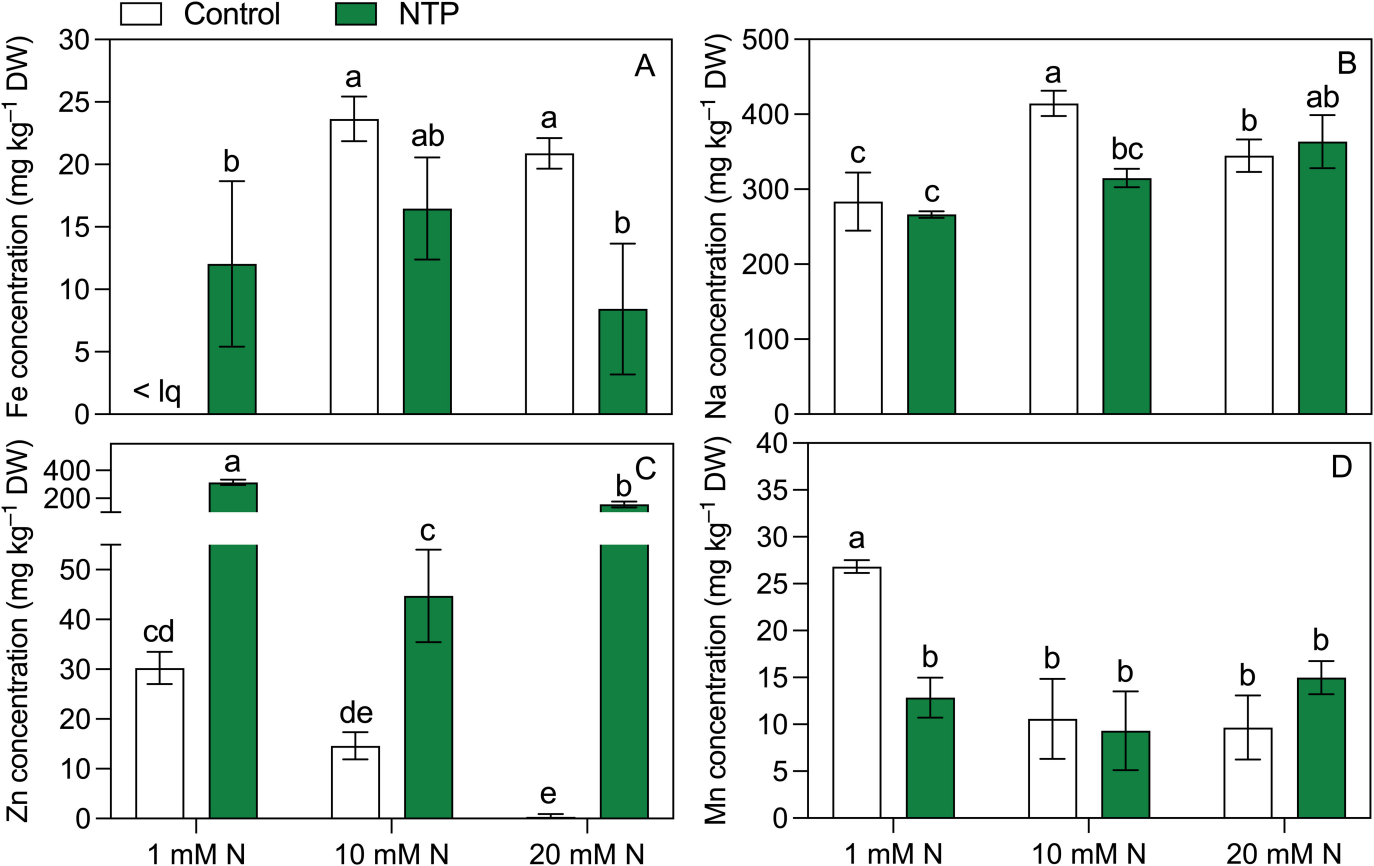
Fe **(A)**, Na **(B)**, Zn **(C)**, and Mn **(D)** concentrations at the 2^nd^ production cycle measured in rocket salad shoot grown using nutrient solution treated or untreated with NTP and with 1, 10, or 20 mM N. Bars represent mean values (n = 4) ± SD. Pairwise comparisons based on Tukey’s *post hoc* are shown in the figure as different letters when two-way ANOVA interaction (N and NTP as factors) was found significant (ANOVA *P*-values are reported in **Table 5**). < lq = below the limit of quantification.

The micronutrient concentrations determined at the 2^nd^ growing cycle showed contrasting trends (**Figure 5**; **Table 5**), but they were generally influenced by NTP and N combination. Iron concentration (**Figure 5A**) was below the quantification limit in control plants (no NTP) fertigated with 1 mM N while it was 12 mg kg^–1^ DW in NTP treated plant under the same N level. An opposite trend was retrieved at 20 mM N, a concentration to which this micronutrient significantly decreased under NTP treatment (– 60%). Sodium concentration (**Figure 5B**) was influenced by both NTP and N concentration. Under control condition, this element increased passing from 1 mM N to 10 mM N while it decreased from 10 mM to 20 mM N. On the contrary, the NTP treatment increased the Na concentration between the 10 mM and 20 mM N levels. Moreover, it decreased in NTP treated plants at 10 mM N compared to the control plants (– 24%). Zinc concentration (**Figure 5C**) was strongly increased by NTP treatment under all N concentrations (+ 938, + 206, + 12470% at 1, 10, and 20 mM N, respectively). This micronutrient was particularly high at 1 and 20 mM N, i.e., 314 and 157 mg kg^–1^ DW in NTP treated rocket plants. Under control conditions (no NTP), Zn concentration decreased by increasing N concentration. Manganese concentration (**Figure 5D**) was higher in shoot of control plants at 1 mM N compared to all other treatments.

## 4 Discussion

In this work the NTP treatment showed contrasting and interaction effects on rocket salad plants in relation to the N concentration in the nutrient solution. Three different levels of N were chosen representing a high, medium, and very low level of N. The first interesting N-dependent effect to note concerns plant yield. The NTP promoted the biomass accumulation at medium concentration (10 mM N), particularly at the 2^nd^ growing cycle that is representative of the marketing product stage, while caused detrimental effects on plant yield at the extreme concentrations (1 and 20 mM N). Indeed, NTP treatments have been found to improve biomass accumulation in several plant species (e.g., Cannazzaro et al., 2021b; Cui et al., 2019; Thirumdas et al., 2018). Despite this, the highest FW and DW were achieved by increasing N concentration, confirming that N fertilization is the prevalent factor involved in stimulating the biomass production. As it is well known, crop yield is indeed positively related to N availability in the root zone. However, when present at high concentration, this element may cause an excess accumulation of NO_3_ in edible organs of leafy vegetables thereby making the product even unmarketable (Massa et al., 2018; Santamaria, 2006). Therefore, simple adding N fertilizers to increase yield may not be the best choice; instead, a proper agronomic strategy should be adopted to find the correct balance between produce yield and quality. Compared with other leafy vegetables, rocket accumulates much higher amount of NO_3_ (Santamaria, 2006), a typical strategy developed by many wild species to counteract the negative effects of osmotic stresses (He et al., 2024). In lettuce, NTP limited the accumulation of NO_3_ (Cannazzaro et al., 2021a). However, to the best of our knowledge, there is no work testing such effects on leafy vegetables under extreme, i.e., very low and very high, or otherwise variable N concentration in the root zone. Our results showed that there is a theoretical limit of N concentration in the root zone above which the NTP treatment would induce detrimental effects on plant physiology. This is likely due to an excess formation and accumulation, in the root zone, of oxidant molecules typically produced under NTP treatment, mainly belonging to the category of RNS, associated to the high level of N. In fact, the NTP-treated water may contain ROS and RNS (Thirumdas et al., 2018) that can be beneficial or harmful for plants, depending on their concentration (Cui et al., 2019). This negative effect of NTP was also observed in case of very long exposure or high intensity treatment of PAW (Cannazzaro et al., 2021a; Nicoletto et al., 2023; Than et al., 2022). Moreover, a higher N concentration in the nutrient solution supplied to rocket plants grown in soilless system has been shown to increase the peroxidase activity and malondialdehyde content, both indicators of oxidative stress (Villani et al., 2023). This suggests that elevated N levels might have had a synergic negative effect with the NTP treatment in promoting oxidative stress. Plants fertigated with 20 mM N and treated with NTP indeed showed a very strong reduction in biomass compared to untreated plants. This result coupled with a F_v_/F_m_ reduction at the 2^nd^ growing cycle highlighting a possible toxic effect on the photosystem II, considering that chlorophyll fluorescence is one of the first parameters affected by oxidative stress (Moustaka and Moustakas, 2023). The negative effect on biomass and photosystem II may have been exacerbated by the cutting and thus more evident at the 2^nd^ productive stage.

A different mechanism appears to have contributed to the biomass reduction under low N. The dry biomass reduction found at 1 mM N in control plants was coupled with a strong increase in anthocyanins. This effect was optically visible by a red colouration of plants under this treatment (data not shown). Anthocyanins are involved in plant response to stress such as N deficiency through well characterized metabolic pathways (Li and Ahammed, 2023); thus, rocket plants not treated with NTP might be more strongly affected by low N level than the NTP treated plants. The stress in these plants might be suggested also by the increase in Mn concentration as a high concentration of this element is usually associated with a higher activity of the antioxidant system (Alejandro et al., 2020) in agreement with the higher anthocyanin content (Gould et al., 2002). The NTP treatment did not increase the concentration of anthocyanins, also under non-limiting N concentration, in contrast with previous results using a red variety of lettuce grown in similar hydroponic conditions in which an increase in these molecules was observed under treatment (Cannazzaro et al., 2021a). In our experimental conditions, the difference in FW between control and NTP treated plants was less evident than the difference in DW because NTP treated plants showed a general higher tissue hydration with lower DW percentage. This is a typical behaviour of plants exposed to NTP treatments, in which a reduced stomatal conductance would play a major role (Cannazzaro et al., 2021b). Nonetheless, NTP treatments has been suggested as a strategy to increase water use efficiency in cropping systems (Brar et al., 2016; Peethambaran et al., 2015), although such practice appears risky under operational conditions. Moreover, NTP treated plants showed a higher K concentration that might stimulate a greater turgor maintenance (Barker and Pilbeam, 2015). The higher DW percentage found in control plants at 1 mM N level compared to the NTP treated plants might be triggered also by the elevated anthocyanin content that has been showed to ensure higher fresh and dry weights under abiotic stress as nutrient deprivation (Yan et al., 2022). Despite the higher DW, control rocket salads had a strong decrease in N and other elements compared with NTP treated plants at 1 mM N. Indeed, the reddish coloration related to anthocyanin overproduction can be also a visual symptom of N deficiency (De Bang et al., 2021). The higher N concentration found in NTP treated plants might be related to the higher NO_3_^−^ availability found in PAW, which would support the hypothesis that NTP treated water can, at least partially, replace conventional N fertilizers thus reducing their supply in agriculture (Ranieri et al., 2021; Thirumdas et al., 2018). However, in our experimental conditions NTP showed peculiar aspects related to the different N level. NTP treated plants increased Kjeldahl N concentration compared to the controls at 1 and 20 mM N and NO_3_ concentration at 1 mM N while in the same plants a decrease in the dry biomass was observed. At the 2^nd^ harvest, all the investigated macrocations increased their concentration in plant tissues of N starved plants irrigated with PAW, which is consistent with the higher accumulation of macroanions (PO_4_ and NO_3_). Similar trends have been observed in the herbaceous organs of floricultural species except for Ca (Cannazzaro et al., 2021b; Traversari et al., 2021). Among the micronutrients, N fertilization had a negative effect on Zn concentrations on NTP untreated plants. Interestingly, this element decreased at increasing N levels while it was particularly high at 1 and 20 mM N levels under NTP treatment. Indeed, the positive effect of NTP treatment on Zn accumulation has been already found in begonia edible flowers (Traversari et al., 2021). The Zn increase particularly evident at 1 and 20 mM N might be also related to the higher level of oxidative stress associated to these NTP treatments as Zn has a well-recognised role in defence mechanisms involved in ROS response (Cakmak, 2000). Calcium, Mg, K, and Zn are essential elements in the human diet and their bioavailability is greater when taken through food rather than synthetic products. Therefore, many works are addressed to obtain biofortified vegetable products (Buturi et al., 2021; White and Broadley, 2009). At the operational level, the most practical and economic technique consists in the supply of high amount of fertilizers at high concentration of the target elements thereby causing their accumulation in edible plants. Fertigation, especially in soilless systems, shows a high efficiency and can be adopted for this purpose (Massa et al., 2023; Sambo et al., 2019). However, it should be highlighted that a massive use of chemical fertilizers may induce plant nutritional imbalance to the detriment of other nutrients. Moreover, above specific limit thresholds in the root zone the residual (unabsorbed) elements can accumulate in the system since the uptake of mineral elements is not proportional to the quantity supplied. These residuals may then accumulate in the environment or require disposal which has environmental and economic implications. Therefore, techniques and technologies that can stimulate the accumulation of nutrients in plants avoiding the use of fertilizers are indeed worth exploring.

In conclusion, the NTP treatment showed different effects related to the N concentration in the nutrient solution. At very low or high concentrations, it had negative effects on biomass. However, if considered the medium N concentration, NTP was able to improve plant biomass production and promote K and Zn accumulation in plant shoots suggesting its suitability for promoting yield and quality of rocket salad plants. Our results highlight also the importance to evaluate the threshold level of N for a crop to avoid the negative effects related to the oxidative stress. Therefore, NTP use is recommended only under an appropriate level of N in nutrient solution which should be carefully evaluated to avoid possible negative effects.

## Data Availability

All relevant data is contained within the article: The original contributions presented in the study are included in the article, further inquiries can be directed to the corresponding author.
